# Compact High-*Q* Bandpass Filter Using 3-D Stacked Stripline

**DOI:** 10.3390/mi17040460

**Published:** 2026-04-09

**Authors:** Yu Cao, Yong Liu, Junling He, Xin Xu

**Affiliations:** 1 School of Electronic Science and Engineering, University of Electronic Science and Technology of China, Chengdu 611731, China; cyeahabs@126.com (Y.C.); jl.he1994@outlook.com (J.H.); 2 Yangtze Delta Region Institute (Huzhou), University of Electronic Science and Technology of China, Huzhou 313001, China; xindereck0512@163.com

**Keywords:** 3-D stacked stripline, T-shaped stepped impedance resonators (SIRs), high-*Q*, compactness, bandpass filter (BPF), transmission zeros (TZs), cross-coupling

## Abstract

This article presents a novel compact high-*Q* bandpass filter (BPF) utilizing a 3-D stacked stripline configuration. T-shaped stepped impedance resonators (SIRs) are employed to achieve miniaturization. By folding the filter geometry from an inline arrangement into a U-shape along the broadside direction, both broadside and edge coupling structures are realized, enabling various cross-coupling schemes for flexible placement of transmission zeros (TZs). A comprehensive analysis of both electric and magnetic coupling structures is conducted to support the overall filter design. To validate the concept, a tenth-order general Chebyshev BPF prototype centered at 3.485 GHz with a 1 dB bandwidth of 380 MHz is designed, fabricated, and measured. The filter is constructed by vertically soldering two patterned sheet metal layers together with three stacked cavities. Despite having an electrical size of only 0.58 × 0.23 × 0.19 *λ_g_*^3^, the filter exhibits a high unloaded *Q*-factor (*Q_u_*) of 1200, along with up to six TZs and a spurious-free frequency range extending to 12 GHz. Measured results show an insertion loss of 0.58 dB at the center frequency and a return loss of better than 20 dB within the passband, demonstrating favorable agreement with simulations. Featuring solid electrical performance, the proposed filter is ideally suited for 5G and 5G-Advanced (5G-A) communication base stations.

## 1. Introduction

As wireless communications evolve from 5G to 5G-Advanced (5G-A) and continue toward 6G, massive multi-input–multi-output (M-MIMO) antennas, as the mainstream form for wireless communication base stations now and for the foreseeable future, are featuring an increasing number of radio frequency (RF) transceiver (TRx) elements [1]. Consequently, the electrical specifications for their embedded preselect filters are becoming increasingly stringent. Due to the relatively high-power consumption and low energy efficiency of current 5G base stations, in sub-6 GHz base station applications, high *Q*-factor or low insertion loss remain the primary considerations for RF filters besides out-of-band rejection. Therefore, complete chip-level integration is not yet feasible, and the current engineering solutions continue to be miniaturized coaxial cavity filters [2] and, over the past decade, an emerging technology—ceramic waveguide (CWG) filters [3,4].

However, both technologies mentioned above have their respective shortcomings. As the traditional filter solution that has been used in wireless communication base stations for decades, coaxial cavity filters offer excellent performance and reliability, validated by millions of base station deployments over many years [2]. Yet, they suffer from large physical volume, and their design optimization has been largely exhausted, making it difficult for them to adapt to the evolving requirements of modern tower-mounted equipment. On the other hand, while CWGs were developed specifically for M-MIMO antennas, their manufacturing processes and material characteristics impose limitations on the flexibility of placing transmission zeros near the passband edge and on spurious suppression in the far stopband [5,6]. This makes it challenging to apply them in scenarios demanding exceptionally stringent near-band rejection, sometimes necessitating a cascaded low-pass filter to meet far-end spurious suppression.

Given this context, achieving a base station filter solution that combines the high electrical performance of traditional cavity filters with the miniaturization advantages of filters like CWG (or any other miniaturized structures) has emerged as a key research focus in recent years. Diverse technologies, such as compact microstrip lines [7,8,9,10] or waveguides [11,12], dielectric waveguide resonators (DWRs) [13,14,15] or monoblock dielectric resonators (MDRs) [16,17,18], suspended microstrip lines [19,20], substrate integrated waveguides (SIWs) [21,22,23,24], substrate integrated suspended lines (SISLs) [25,26], low temperature cofired ceramic (LTCC) structures [27,28], and empty substrate integrated coaxial line (ESICL) [29], etc. Among these reports, multilayer configurations like LTCC, SISL, and SIW offer significant miniaturization advantages, but at the cost of reduced *Q*-factor due to increased dielectric loss. How to significantly enhance the *Q*-factor by combining cavity structures with the abovementioned multilayer configurations presents a compelling research direction.

In this paper, a compact high-*Q* bandpass filter (BPF) based on a 3-D stacked stripline configuration is presented. A T-shaped stepped impedance resonator (SIR) is incorporated to not only miniaturize the resonator but also to extend the frequency range of spurious suppression. By folding the filter topology from a conventional inline layout into a compact U-shape along the broadside direction, the design facilitates the realization of both broadside and edge coupling mechanisms. This 3-D arrangement is pivotal, as it enables the implementation of various cross-coupling schemes, enhanced flexibility in generating and positioning transmission zeros (TZs) to improve out-of-band rejection; meanwhile, resulting in a compact overall filter configuration. A comprehensive theoretical analysis of the distinct electric and magnetic coupling structures integral to this configuration is presented to guide the filter synthesis. To experimentally validate the proposed concept, a rigorous tenth-order general Chebyshev BPF prototype is designed, fabricated, and characterized. Measured results match the simulations very well and demonstrate an encouraging combination of compactness and performance.

## 2. Analysis of T-Shaped SIR

**Figure 1:**
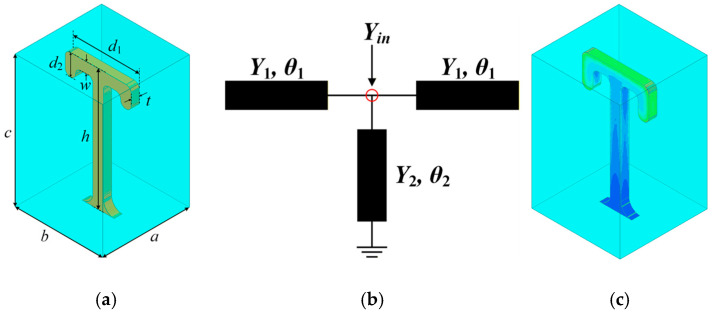
(**a**) Schematic structure of the resonator; (**b**) Equivalent transmission line model; (**c**) Electric field distribution of the resonator.

Figure 1a illustrates the schematic structure of the proposed resonator, which features a stripline composed of a patterned layer and a metal cavity. For the miniaturization of the resonator, a T-shaped stub is loaded at the open-end to form an SIR, and its equivalent transmission line model is shown in Figure 1b. The left and right horizontal arms can be modeled as two sections of short-circuited stub transmission lines (characteristic admittance *Y*_1_; electrical length *θ*_1_). The central vertical arm can be modeled as a section of an open-circuited stub transmission line (characteristic admittance *Y*_2_; electrical length *θ*_2_). When the input port is defined at the T-shaped connection node to calculate the equivalent input admittance *Y*_in_, its value can be derived as follows:

(1) $$Y_{\mathrm{in}}=2jY_{2}\tan\theta_{2}-jY_{1}\cot\theta_{1}$$

where *θ_i_* = *βl_i_*, *β* = 2*πf*/*c* is the phase constant, and *l_i_* is the physical length of one of the stubs. The resonant frequency *f*_0_ can be obtained from *Y*_in_ = 0:

(2) $$2Y_{2}\tan\left(\frac{2\pi f_{0}}{c}l_{2}\right)=Y_{1}\cot\left(\frac{2\pi f_{0}}{c}l_{1}\right)$$

Like the classical SIR, the characteristic impedance ratio of this resonator also carries meaningful physical implications, and it can be denoted as follows:

(3) $$k=\frac{Z_{2}}{Z_{1}}=\frac{Y_{1}}{Y_{2}}$$

where *Z*_1_ and *Z*_2_ are the characteristic impedances of short-circuited and open-circuited stubs, respectively. Substituting Equation (2) into Equation (3), the ratio of this structure is obtained as follows:

(4) $$k=2\tan\theta_{1}\tan\theta_{2}$$

This is a crucial formula that governs the harmonic frequencies of this resonator. A larger *k* shifts the first harmonic to a higher frequency, vice versa. To highlight the improved advantages of the proposed resonator structure, Table 1 compares it with the conventional SIR. It indicates that when having the same electrical length, the T-shaped SIR has a better harmonic suppression performance than the conventional one. Conversely, when having equal characteristic impedance, the T-shaped one has a smaller size than the conventional one.

Figure 1c illustrates the electric field distribution of the resonator. It is seen that the electric field concentrates on the open-circuited side, enhancing capacitive loading to ground and thus further reducing the electrical length. Moreover, the end parts of both open-circuited stubs are eventually parallel to the short-circuited section. By distributing the electrical lengths between the horizontal and vertical parts of these stubs, the coupling to adjacent resonators can be adjusted more easily, leading to higher design flexibility.

**Figure 2:**
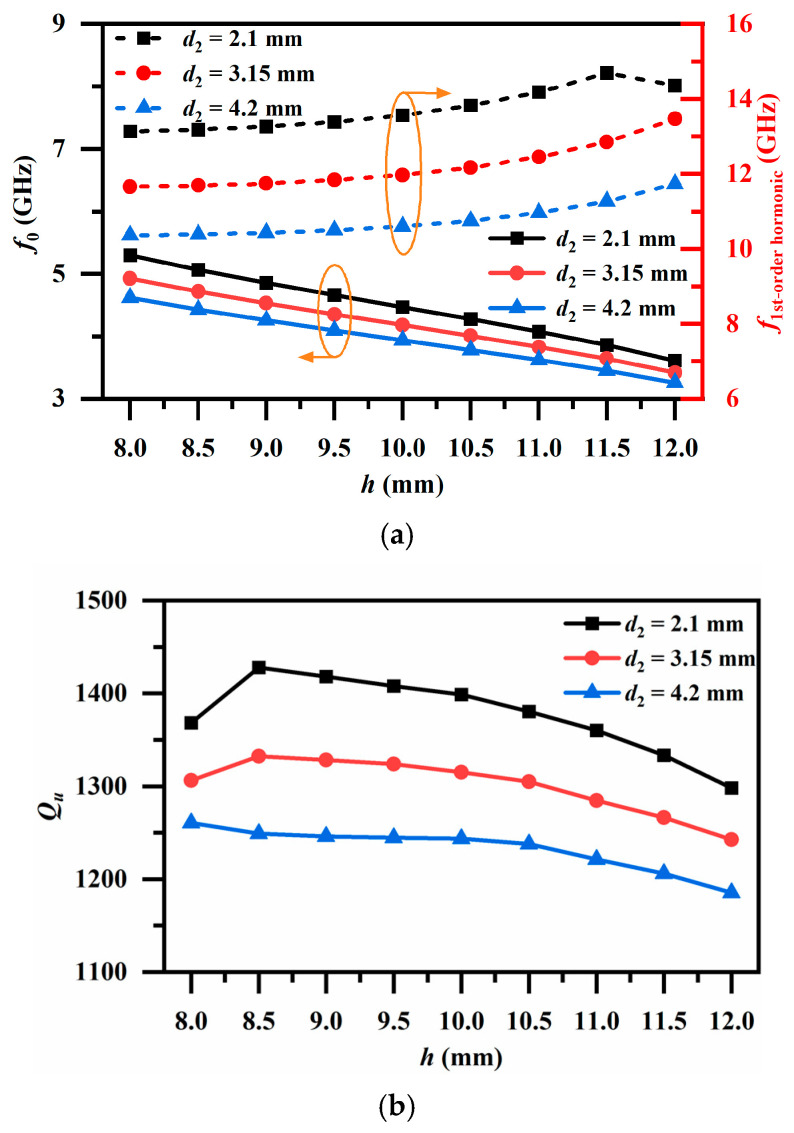
Resonant characteristics of the resonator. Under different *d*_2_ conditions: (**a**) variations in the fundamental mode and first higher-order mode resonant frequencies, and (**b**) variation in the fundamental mode *Q_u_* with *h*.

Full-wave electromagnetic simulation tool ANSYS HFSS (Ansys Electronics Desktop 2022 R1) is employed for further numerical analysis. Figure 2a illustrates the variations in the fundamental mode resonant frequency *f*_0_ and the first higher-order mode resonant frequency *f*_1_ with the cavity height *h* under different *d*_2_ conditions. It can be observed that as *h* increases, *f*_0_ exhibits an overall monotonic downward trend; in contrast, *f*_1_ exhibits an upward trend, which means that if dimensions permit, a longer *h* can achieve a wider spurious-free frequency range. However, miniaturization is a main target of the proposed design; thus, constraints must be placed on the resonator length. An extended study is conducted on the folded section length *d*_2_. The results indicate that as *d*_2_ increases, the fundamental frequency *f*_0_ experiences a slight decrease, whereas the first spurious frequency *f*_1_ is significantly reduced. This demonstrates that a trade-off consideration needs to be made between “more compact” and “wider spurious-free frequency range”. Therefore, by jointly adjusting *h* and *d*_2_, the fundamental and higher-order mode positions can be configured respectively, thereby accommodating miniaturized requirements while ensuring the distribution of a spurious-free frequency range.

Figure 2b presents the variation trend of the unloaded quality factor (*Q_u_*) with respect to *h*. Under different *d*_2_ conditions, *Q_u_* decreases slowly as *h* increases, and a larger *d*_2_ corresponds to a lower *Q_u_*, which means another compromise element needs to be considered with resonator size. It should be noted that within the swept parameter range, *Q_u_* remains at a relatively high level. This demonstrates that the T-shaped SIR can maintain good high-*Q* characteristics while achieving low-frequency tuning, providing a guarantee of high *Q_u_* for the subsequent implementation of multi-resonator coupled filters. With this, the geometric parameter settings for the proposed resonator are detailed in Table 2.

## 3. Compact High-Q BPF Design

### 3.1. Filter Synthesis and 3-D Stacked Configuration

**Figure 3:**
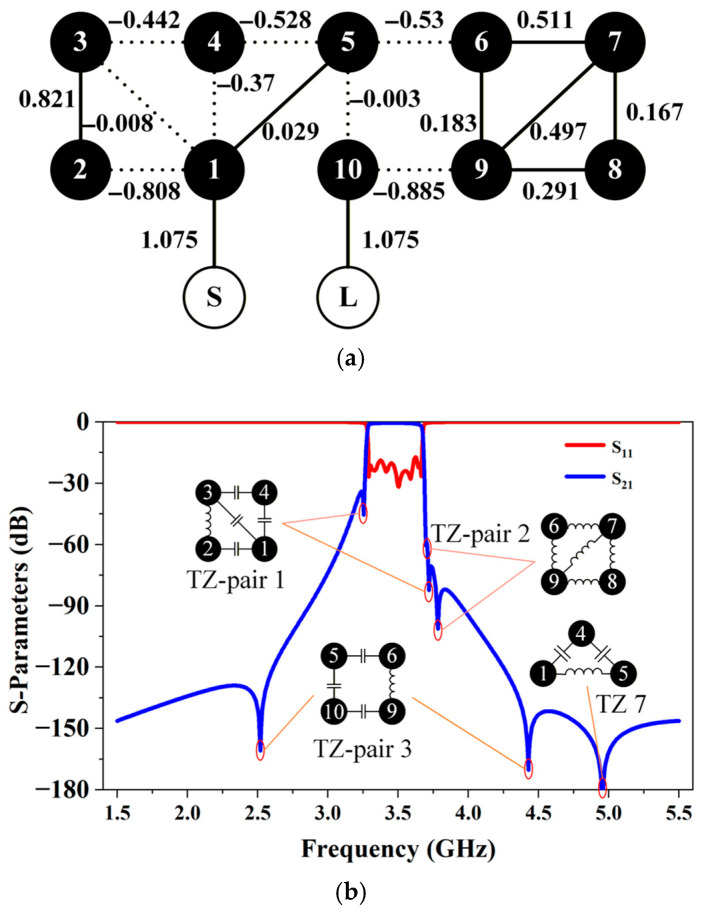
(**a**) BPF topology and normalized coupling coefficients; (**b**) Synthesized ideal response by introducing cross-coupling structures.

To demonstrate the proposed 3-D stacked concept, a tenth-order generalized Chebyshev BPF has been designed. This filter operates at 3.485 GHz with a 1 dB bandwidth of 380 MHz. To validate the flexible TZ controllability of the filter, four TZs are deployed near the passband edges at 3.25, 3.7, 3.72, and 3.76 GHz, respectively. Figure 3a illustrates the topology and synthesized results of the filter, where solid lines indicate magnetic coupling, and dotted lines represent electric coupling. Figure 3b displays two TZ-pairs (1 and 2) with the corresponding cascaded quadruplet (CQ) structures. In addition, three additional TZs (pair 3 and TZ 7) are introduced to improve the far out-of-band rejection. Notably, such a TZ strategy, along with the complex topology, is overly idealized, making its physical implementation challenging. The structural scheme proposed in this paper can reasonably map and realize all the coupling relationships required by this topology.

**Figure 4:**
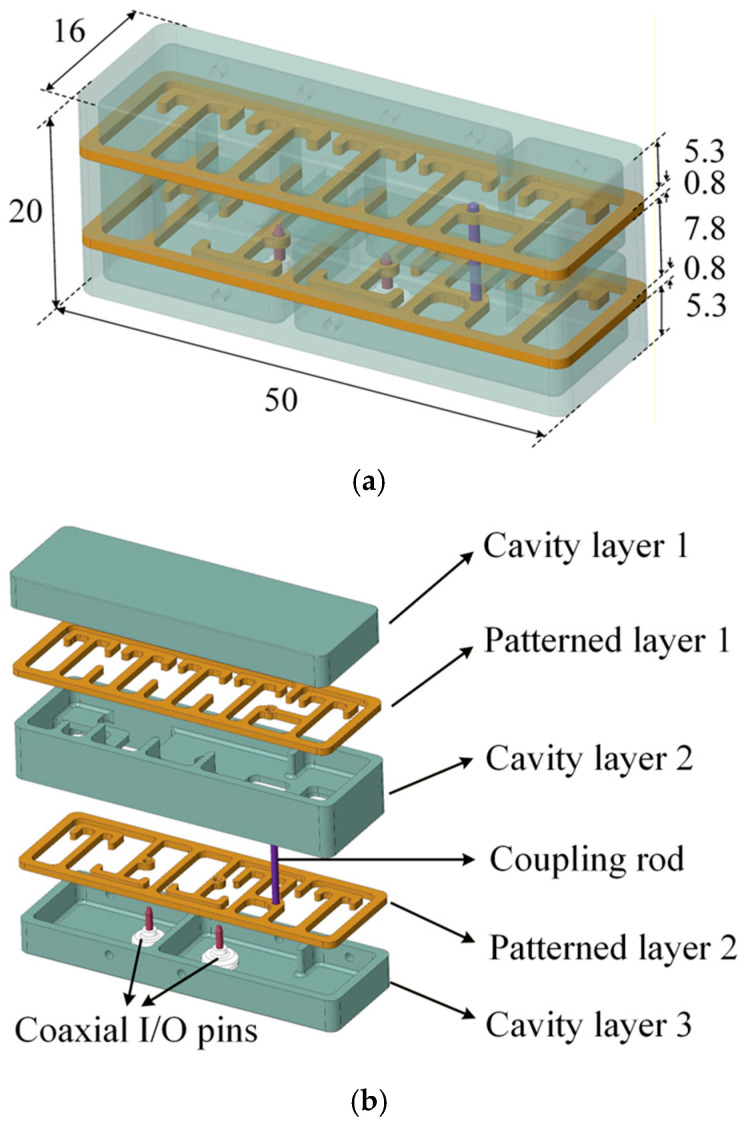
(**a**) Profile (Unit: mm) and (**b**) exploded view of the 3-D stacked BPF.

Figure 4a depicts the profile of the proposed filter, which consists of three cavity layers, two metal sheet patterned layers, and two input/output (I/O) pins stacked vertically, as presented in Figure 4b. All the layers are soldered to each other to ensure low impedance Ohmic contact and tight electromagnetic shielding. Compared to traditional planar structures, the 3-D stacked form facilitates the introduction of the vertical dimension as an additional degree of freedom, and more coupling paths between resonators can be flexibly incorporated within a compact room that incurs only a minimal increase in height. Two coaxial connectors are riveted at the bottom cavity, enabling a surface-mount configuration that simplifies integration with the system motherboard.

### 3.2. Coupling Mechanisms and Analysis

To accurately translate the target coupling relationships from topology synthesis into physical design, various coupling structures are analyzed and incorporated. According to Reference [30], the external quality factor *Q_e_* and the coupling coefficient *k_ij_* can be expressed as follows:

(5) $$Q_{e}=\frac{1}{FBW\times M_{S1}^{2}}$$

(6) $$k_{ij}=FBW\times M_{ij}$$

where *FBW* is the fractional bandwidth, *M_S_*_1_ and *M_ij_* are the elements of the coupling matrix. Their values have been synthesized and are shown in Figure 3a. The theoretical target values of *Q_e_* and *k_ij_* calculated from these expressions are summarized in Table 3. The corresponding practical values of the physical structures, obtained via EM-simulation tools, can be expressed as follows:

(7) $$Q_{e}=\frac{\omega_{0}\tau_{\max}}{4}$$

(8) $$k=\frac{f_{2}^{2}-f_{1}^{2}}{f_{2}^{2}+f_{1}^{2}}$$

where *ω*_0_ represents the angular resonance frequency, *τ_max_* is the maximum group delay of *S*_11_, and *f*_1_ and *f*_2_ are the two resonant frequencies generated after the coupling of the two resonators. By sweeping key geometric parameters, curves of *k_ij_* varying with dimensions can be obtained. This establishes a quantitative mapping between the target *k_ij_* and physical dimensions, providing a reference basis for the structural realization and dimension derivation of each coupling element. Next, we provide a detailed analysis of the different coupling structures utilized in this filter.

**Figure 5:**
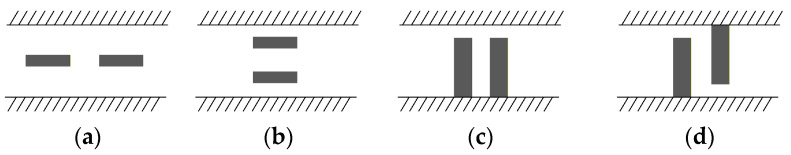
Four fundamental coupling configurations of (**a**) edge coupling, (**b**) broadside coupling, (**c**) coupling via identically oriented resonators, and (**d**) coupling via oppositely oriented resonators.

Figure 5 illustrates the basic coupling configurations applicable to this filter. Edge coupling in the horizontal direction mainly provides sequential coupling between resonators, while broadside coupling in the vertical direction primarily enables cross-coupling. Unlike planar multilayer technologies like SISL or LTCC, the all-metal-layer 3-D stacked structure used in this filter results in air-filled standard stripline resonators, rather than dielectric-filled or semi-air-semi-dielectric structures on dielectric substrates. Consequently, the proposed resonator exhibits a higher *Q*-factor, and the distance to the ground planes can be flexibly adjusted without being limited by the standard thickness constraints of PCB, prepreg, or LTCC green tape.

**Table 3:** Values of external quality factor and coupling coefficients.

Sequential Coupling			External Quality Factor *Q_e_* (*Q_S_*_1_ or *Q*_10*L*_)		
*k*_12_	*k*_23_	*k*_34_	7.94		
−0.088	0.090	−0.048	Cross-coupling		
*k*_45_	*k*_56_	*k*_67_	*k*_13_	*k*_14_	*k*_15_
−0.058	−0.058	0.056	−0.0009	−0.040	−0.003
*k*_78_	*k*_89_	*k*_9-10_	*k*_5-10_	*k*_69_	*k*_79_
0.018	0.032	−0.097	−0.0003	0.020	0.054

Figure 5c,d show the two edge-coupling arrangements mainly used in this filter. For the arrangement of resonators in the same orientation, if electric coupling is desired, the open-circuited sides of the two resonators should be brought close together, and the short-circuited sides between them should be properly shielded; the opposite holds for magnetic coupling. When arranged in reverse, the high-current-density regions of the two resonators are inversely positioned, making it difficult for their magnetic fields to interlink; thus, the magnetic coupling is weak. In contrast, this arrangement results in a large potential difference between the open and short ends of the two resonators, producing strong electric coupling. Consequently, the overall coupling appears capacitive, and its strength can be conveniently controlled by adjusting the spacing between the resonators. These arrangements also apply to broadside coupling. No matter which arrangement is employed, when stronger coupling is required, the two resonators can be interconnected by means of a coupling loop, a coupling rod, or similar structures.

#### 3.2.1. Edge Coupling Structures

**Figure 6:**
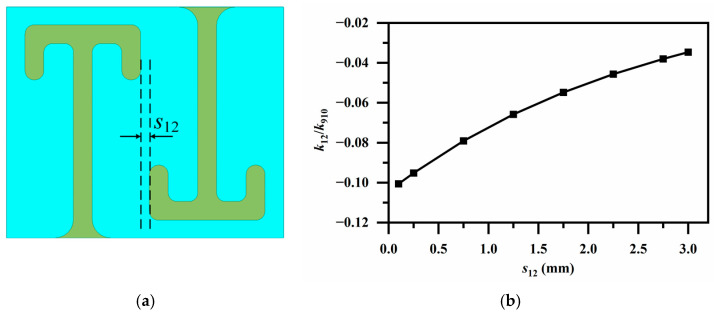
(**a**) Electric coupling via oppositely oriented resonator pair (for *k*_12_ and *k*_910_); (**b**) Variation in coupling coefficients *k_ij_* with resonator spacing *s*_12_.

**Figure 7:**
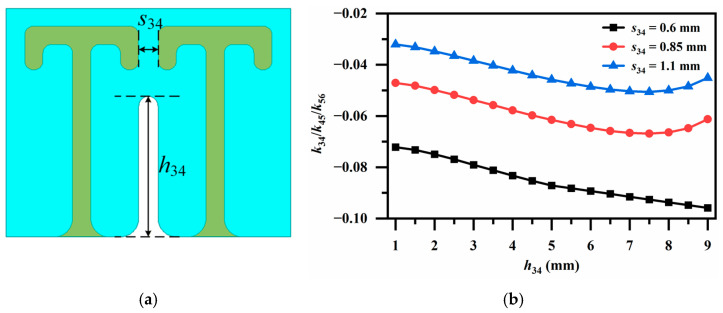
(**a**) Electric coupling via identically oriented resonator pair (for *k*_34_, *k*_45_ and *k*_56_); (**b**) Variation in coupling coefficients *k_ij_* with aperture height *h*_34_ under different *s_34_* conditions.

**Figure 8:**
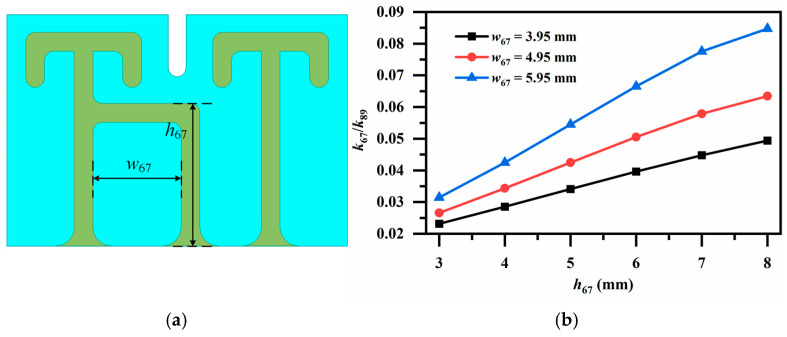
(**a**) Magnetic coupling via coupling loop (for *k*_67_ and *k*_89_); (**b**) Variation in coupling coefficients *k_ij_* with coupling loop height *h*_67_.

Figure 6, Figure 7 and Figure 8 illustrate all edge coupling structures employed in this filter design. An oppositely oriented resonator pair is chosen for strong electric coupling, *k*_12_ and *k*_910_, as shown in Figure 6a. A wide coupling range is readily obtained by tuning the spacing *s*_12_ between the resonator pair in Figure 6b.

The coupling structure for *k*_34_, *k*_45_ and *k*_56_ employs identically oriented resonator pairs shown in Figure 7a. As aforementioned, to achieve strong enough electric coupling, the coupling window is placed at the open-circuited end, whereas the short-circuited end is shielded by a metal strip to minimize the cancelation of the electric coupling by the magnetic coupling. As shown in Figure 7b, varying the spacing *s*_34_ between the open-circuited stubs of the two resonators leads to a significant change in coupling strength. Further fine-tuning can be achieved by adjusting the height *h*_34_ of the metal strip, ultimately yielding the desired coupling coefficient.

In Figure 8a, a coupling loop is introduced between the resonators where a magnetic coupling needs to be strengthened. By adjusting the width and height of the coupling loop, the coupling strength can be flexibly tuned. The final configuration corresponding to the desired magnetic coupling can be obtained in Figure 8b.

#### 3.2.2. Broadside Coupling Structures

**Figure 9:**
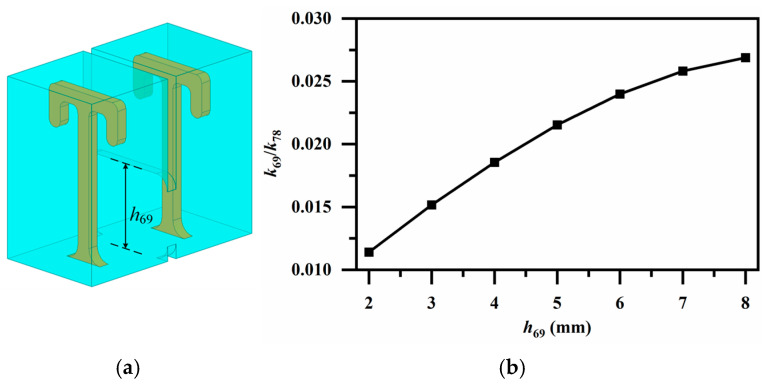
(**a**) Magnetic coupling via aperture with identically oriented resonator pair (for *k*_69_ and *k*_78_); (**b**) Variation in coupling coefficients *k_ij_* with aperture height *h*_69_.

**Figure 10:**
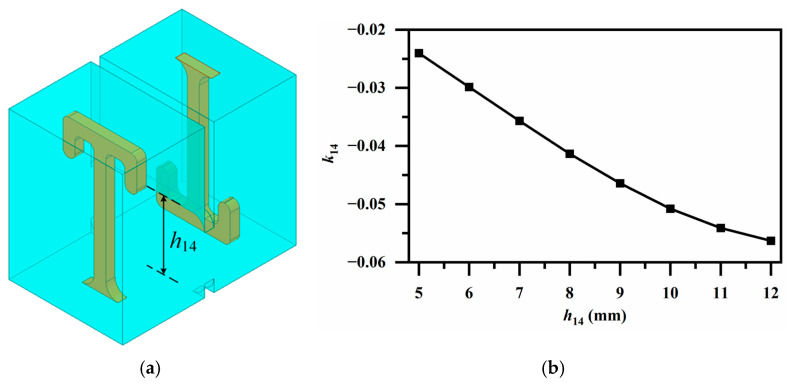
(**a**) Electric coupling via aperture with oppositely oriented resonator pair (for *k*_14_); (**b**) Variation in coupling coefficient *k*_14_ with aperture height *h*_14_.

**Figure 11:**
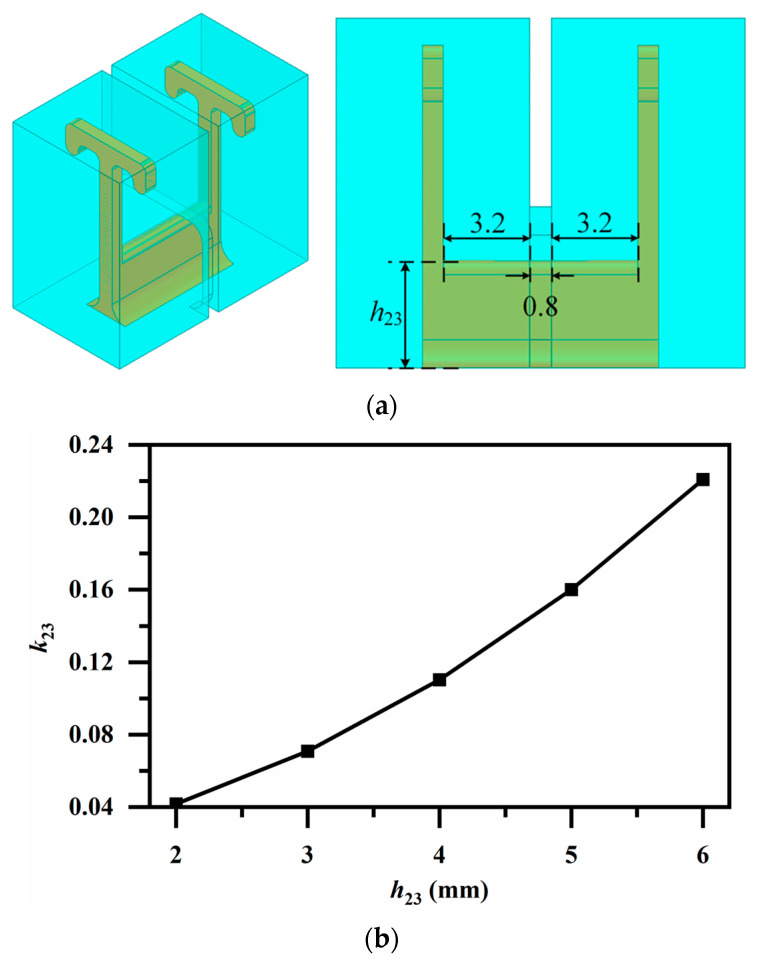
(**a**) Magnetic coupling via ridge (for *k*_23_) (Unit: mm); (**b**) Variation in coupling coefficient *k*_23_ with ridge height *h*_23_.

Figure 9, Figure 10 and Figure 11 illustrate employed broadside coupling structures, introduced by a 3-D stacked configuration. In Figure 9a, an aperture placed between the two short-circuited ends provides moderate magnetic coupling between identically oriented resonator pairs on different patterned layers, realizing *k*_69_ and *k*_78_, as depicted in Figure 9b. Similarly, the aperture between the oppositely oriented resonator pair offers electric coupling for *k*_14_ as shown in Figure 10. A ridge coupling structure is introduced to obtain strong magnetic coupling in Figure 11a. It can be observed from Figure 11b that, while keeping other dimensions constant, adjusting the height of the ridge provides a wide tuning range and sufficient magnetic coupling, thereby achieving the required coupling strength for *k*_23_.

#### 3.2.3. Hybrid Edge and Broadside Coupling Structures

**Figure 12:**
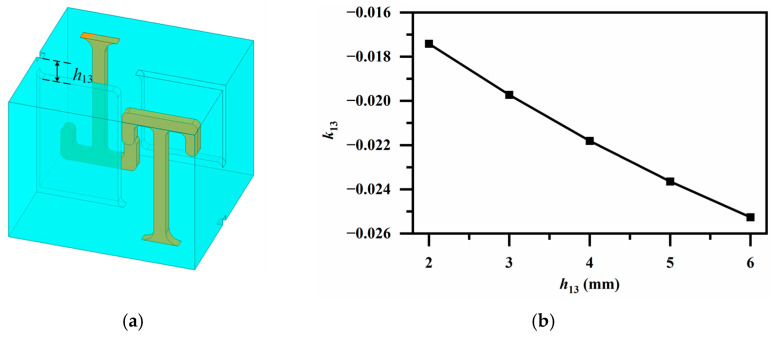
(**a**) Electric coupling via oblique aperture with oppositely oriented resonator pair (for *k*_13_); (**b**) Variation in coupling coefficient *k*_13_ with aperture height *h*_13_.

**Figure 13:**
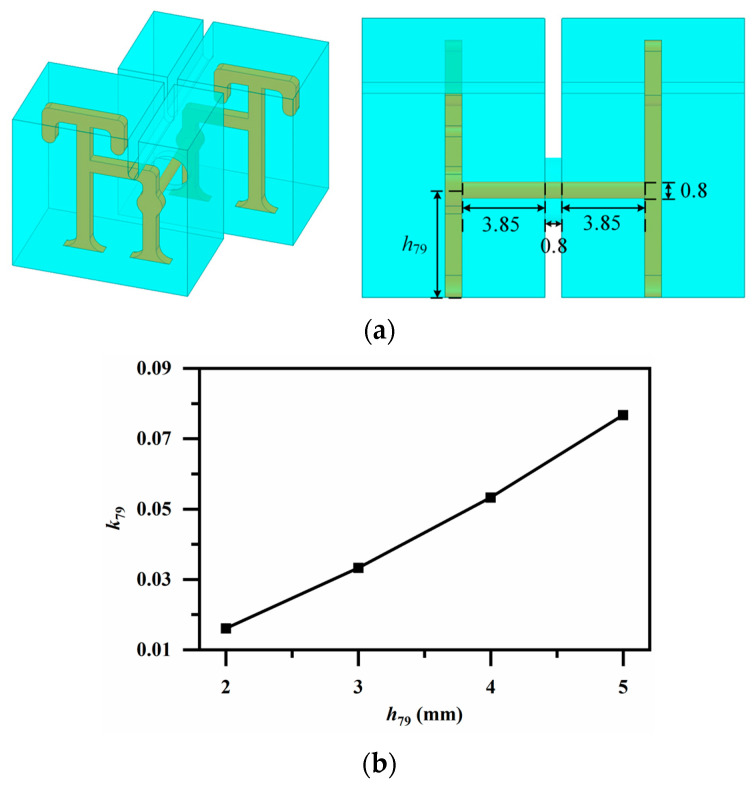
(**a**) Magnetic coupling via rod (for *k*_79_) (Unit: mm); (**b**) Variation in coupling coefficient *k*_79_ with coupling rod height *h*_79_ (the upper end represents the center position of the cylindrical rod).

Some cross-coupling implementations require a hybrid coupling scheme combining both edge and broadside coupling, as illustrated in Figure 12 and Figure 13. An oblique aperture is adopted between oppositely oriented resonator pairs in Figure 12a; thus, an electric coupling relationship shown in Figure 12b is obtained for *k*_13_.

The implementation of *k*_79_ poses a challenge because the two resonators reside on different patterned layers and cannot be directly aligned. To address this, a coupling loop is incorporated into each resonator, with the loops directly connected by a vertical rod, as depicted in Figure 13a. The required magnetic coupling is then easily obtained by tuning the rod height, as demonstrated in Figure 13b.

Overall, taking advantage of the innovative 3-D stacked structure, numerous coupling configurations are implemented to readily realize the desired complex Chebyshev response topology.

### 3.3. I/O Port Design

**Figure 14:**
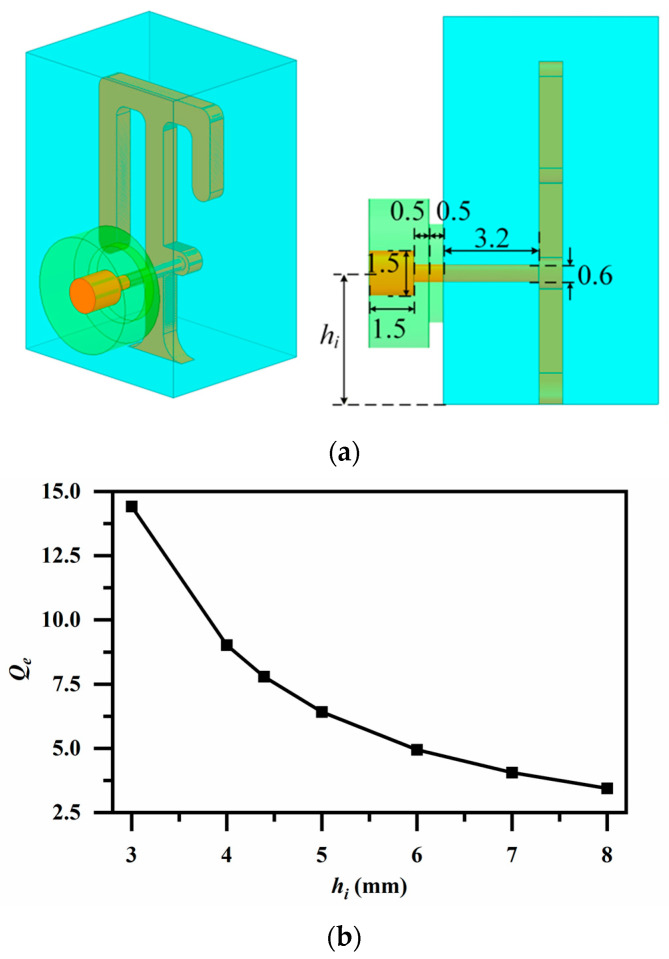
(**a**) Structure of the I/O port (Unit: mm); (**b**) Variation in loaded *Q_e_* value with input pin height *h_i_*.

The I/O port structure of the proposed filter is shown in Figure 14a. A coaxial pin connector is employed to meet the surface-mount requirement. The dielectric material of the connector is polytetrafluoroethylene (PTFE). To ensure proper matching, a single-step impedance transition is implemented in both the dielectric support and the inner conductor along the radial direction. Then, the external *Q*-factor (*Q_e_*) of the I/O port is determined by the insertion height of the pin perpendicular to the first resonator *hᵢ*, and their quantitative relationship is illustrated in Figure 14b. Notably, the single-arm open-circuited stub of the first resonator can be optimized in conjunction with the pin insertion position to achieve the optimal *Qₑ*.

**Figure 15:**
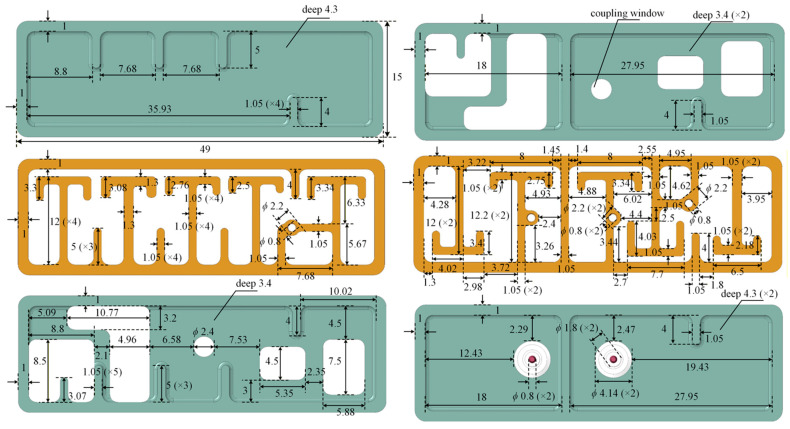
Layer-by-layer structural details and corresponding dimensions (Unit: mm) of the 3-D stacked BPF.

Based on the design of the aforementioned individual parts, the overall filter model is optimized and simulated. The final structural design of each metal layer, along with its detailed dimensions, is shown in Figure 15. It is worth noting that a coupling window with a diameter of 2.4 mm is introduced in the intermediate cavity layer to achieve weak coupling *k*_510_, thereby forming a CQ structure and resulting in TZ pair 3 in Figure 3b. Additionally, a weak coupling (*k*_15_) is established between resonators 1 and 5 via the coupling window intended for *k*_14_. This formed a cascaded triplet (CT) structure, subsequently yielding TZ 7. These three transmission zeros are located farther from the passband and are primarily intended to enhance out-of-band rejection. At this point, the design of all cross-coupling structures is complete.

## 4. Fabrication and Measurement

**Figure 16:**
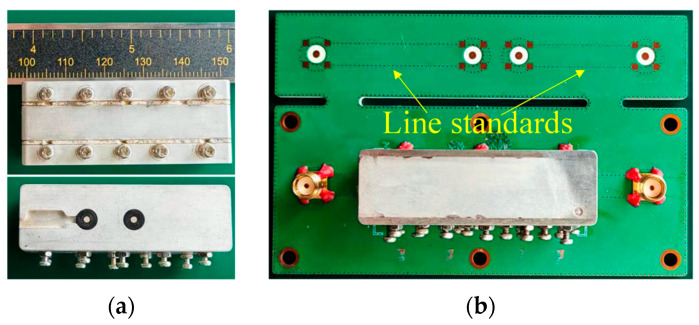
Photographs of (**a**) prototype filter, and (**b**) testing fixture.

The proposed filter is fabricated as shown in Figure 16a. The two sheet metal stripline layers are fabricated from stainless steel using wire electrical discharge machining (EDM) with a tolerance of ±20 μm, while the three cavity layers are machined from aluminum alloy using computer numerical control (CNC) processing with a tolerance of ±30 μm. All metal layers are plated with 5 μm copper and then 3 μm silver, and subsequently assembled vertically by soldering using a dedicated assembly soldering fixture to ensure vertical alignment accuracy of less than ±30 μm. The bottom coaxial pin connectors are riveted to the bottom cavity layer using a dedicated fixture to ensure tight contact. The overall dimensions of the filter are 50 × 20 × 16 mm^3^. Tuning screws are placed on the side wall of the filter above each resonator, aligned parallel to them, to compensate for frequency shifts caused by fabrication and assembly errors. The coupling between resonators is ensured by machining precision, thus eliminating the need for tuning. Figure 16b shows the test fixture of the filter. By de-embedding the line standards and subminiature version A (SMA) connectors on the PCB board, the additional insertion loss introduced by the test fixture can be calibrated.

**Figure 17:**
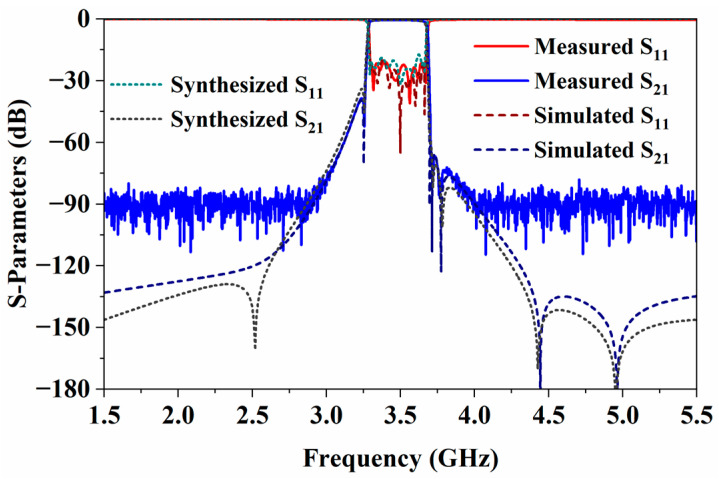
Synthesized, simulated, and measured results of the filter.

**Figure 18:**
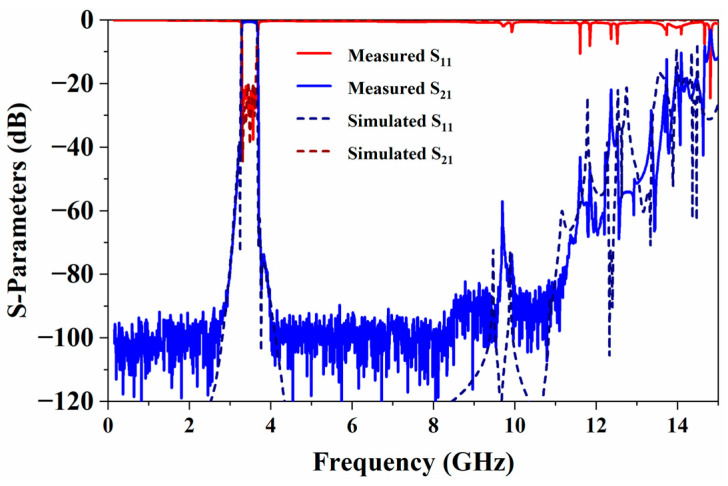
Simulated and measured results in a wide frequency range.

The comparison among the synthesized, simulated, and measured results of the proposed BPF is shown in Figure 17. The filter exhibits excellent in-band electrical performance, with an insertion loss of 0.58 dB at the center frequency and a return loss better than 20 dB across the entire passband, while measured curves match the simulated and synthesized results very well. The TZs near the passband edges are clearly visible in both the simulation and measurement, and their frequency locations are highly consistent with the synthesized results, demonstrating the controllability and precision of the TZs introduced by cross-coupling. For the far-band TZs, it can be observed that the simulated results are also in good agreement with the synthesized ones. Although submerged in the noise floor in the measurement results, they effectively enhance the far-band rejection capability of the filter. The only discrepancy is that a TZ in the synthesized results at 2.5 GHz in the lower stopband does not appear in the simulation. A thorough examination of the filter’s EM simulation model reveals that the coupling window between resonators 5 and 10 also introduces a very weak magnetic coupling *k*_59_ between resonators 5 and 9 (about 0.001). This parasitic coupling is exactly what cancels the transmission zero at 2.5 GHz. Thus, this filter ultimately exhibits six TZs. Figure 18 illustrates the wideband frequency response of the filter. Benefiting from the T-shaped SIR, the filter exhibits excellent spurious suppression performance, with an upper stopband rejection greater than 40 dB up to 12 GHz (3.45*f*_0_).

A comprehensive comparison has been conducted between the proposed filter and various state-of-the-art miniaturized filters reported in recent years in Table 4. The results demonstrate that the proposed filter achieves a good performance trade-off, with overall behavior that is comparatively favorable. Specifically, its *Q*-factor and insertion loss are comparable to those of advanced miniaturized waveguide technologies. Simultaneously, its physical size is comparable to that of various 3-D and planar miniaturized structures. Furthermore, the proposed filter offers distinct advantages in terms of TZ placement and satisfactory spurious suppression performance.

## 5. Conclusions

A new kind of compact high-*Q* BPF based on a 3-D stacked stripline configuration is presented in this paper. By employing T-shaped SIR, the resonator size is appreciably reduced while maintaining a high *Q_u_* of up to 1200, simultaneously achieving improved spurious suppression. The innovative multilayer metal stacking scheme enables a compact U-shaped geometry in the broadside direction, facilitating the easy realization of various coupling schemes, such as edge coupling, broadside coupling, and their hybrid forms. This approach effectively satisfies the ideal topology requirements of complex generalized Chebyshev functions. Consequently, in this tenth-order filter, six TZs are accurately placed through various forms of cross-coupling, considerably enhancing both the near-band and far-out-of-band rejection performance. A filter prototype has been designed, fabricated, and characterized. Measured results validate its favorable electrical performance, highlighted by low in-band insertion loss, sharp near-band rejection, and wideband spurious suppression. The filter also features a compact footprint compatible with surface-mount technology (SMT). With a well-optimized design trade-off compared to state-of-the-art miniaturization techniques, it is ideally suited for high-performance applications, including M-MIMO 5G/5G-A base transceiver stations and satellite communication systems. This filter also shows favorable application potential for phased-array antenna systems with high transmission power or high receiving sensitivity and large numbers of transceiver channels, such as radio telescope or detection radar arrays.

## Figures and Tables

**Table 1:** Comparison between the T-shaped and conventional SIRs.

Performance	T-Shaped	Conventional	Notes
Structure	Single short-circuited with two symmetric open-circuited stubs	Single short-circuited with single open-circuited stubs	One more degree of freedom
Resonant condition	*k* = 2tan*θ*_1_tan*θ*_2_	*k*′ = tan*θ*_1_′tan*θ*_2_′	Bigger scale factor of 2
*k* with *θ*_1_ = *θ*_2_ (*k*′ with *θ*_1_′ = *θ*_2_′)	*k* = 2tan^2^*θ*	*k*′ = tan^2^*θ*′	*k* > *k*′ when *θ* = *θ*′
*θ* with *θ*_1_ = *θ*_2_ (*k*′ with *θ*_1_′ = *θ*_2_′)	$\theta =\arctan\sqrt{k/2}$	$\theta \prime =\arctan\sqrt{k’}$	*θ* < *θ*′ when *k* = *k*′

**Table 2:** Geometric parameter settings of the resonator (Unit: mm).

Parameter	*a*	*b*	*c*	*h*	*t*	*w*	*d*_1_	*d*_2_
Value	8.5	8.6	13	12	0.8	1.05	6.5	2.1

**Table 4:** Comparison between the proposed filter and other miniaturized designs.

Ref.	Tech.	*f*_0_ (GHz)	FBW (%)	RL (dB)	IL (dB)	*Q_u_*	Order	NTZs/ CNTZs	Spurious (×*f*_0_)	Volume (*λ*_g_^2^ or *λ*_g_^3^)
[9]	Microstrip	0.97	25.7	11.8	3.4	380	6	2/2	10	0.097 × 0.103
[11]	Waveguide	5	8	20	0.5	2230	5	3/2	1.5	0.673 × 0.337 × 1.433
[14]	Dielectric waveguide	3.5	5.7	18	1	625	4	2/2	1.2	0.42 × 0.21 × 0.05
[16]	Monoblock dielectric	2.597	6.35	18	1.55	1000	10	2/2	2.1	1.14 × 0.84 × 0.22
[19]	Suspended stripline	0.67	23.8	19.5	0.38	1075	5	1/1	2.2	0.31 × 0.158
[22]	SIW	0.92	22.1	16	0.83	226	4	0	3.98	0.15 × 0.3
[23]	SIW	3.48	16	16	1	NG	4	4/2	2.37	0.18 × 0.14 × 0.02
[25]	SISL	3.45	18.8	20	1.63	NG	4	2/1	2.6	0.45 × 0.29
[26]	SISL	1.83	11	15	1.91	359	4	0	2.77	0.038 *
[27]	LTCC	5	6.76	8.2	3.48	160	4	2/2	1.6	0.72 × 0.37
T.W.	3-D stacked stripline	3.485	11	20	0.58	1200	10	6/6	3.45	0.58 × 0.23 × 0.19

T.W.: This work; * Unit: *λ*_g_^2^; Tech.: Technology; NTZs: Number of TZs; CNTZs: Number of controllable TZs.

## Data Availability

The original contributions presented in this study are included in the article. Further inquiries can be directed to the corresponding author.
